# Aaqueous exposure to silver nanoparticles synthesized by abalone viscera hydrolysates promotes the growth, immunity and gut health of zebrafish (*Danio rerio*)

**DOI:** 10.3389/fmicb.2022.1048216

**Published:** 2022-12-08

**Authors:** Jing Ni, Zhuan Yang, Yue Zhang, Ying Ma, Hejian Xiong, Wenjie Jian

**Affiliations:** ^1^Fisheries College, Key Laboratory of Healthy Mariculture for the East China Sea, Ministry of Agriculture and Rural Affairs, Jimei University, Xiamen, China; ^2^College of Ocean Food and Biological Engineering, Jimei University, Xiamen, China; ^3^Xiamen Medical College, Institute of Respiratory Diseases, Xiamen, China

**Keywords:** silver nanoparticles, gut microbiota, growth performance, antioxidation, immunity, zebrafish

## Abstract

Silver nanoparticles (AgNPs) have the potential to be used in aquaculture, but their influence on the growth and health of aquatic organisms has not been extensively investigated. In this study, the abalone viscera hydrolysates decorated AgNPs (AVH-AgNPs) were dispersed into aquaculture water at different concentrations (0, 6, 9, and 18 μg/l) to evaluate the biological effects on zebrafish (Danio rerio). The results showed that the AVH-AgNPs treatments of 6 and 9 μg/l promoted the growth and did not cause obvious damage to the gills, intestines, and livers of zebrafish. All the treatments induced catalase (CAT) and superoxide dismutase (SOD) activities and increased glutathione (GSH) content in the livers and upregulated the expression of immune related genes. The effects of 9 and 18 μg/l AVH-AgNPs treatments were more obvious. After AVH-AgNPs treatment, the abundances of some potential pathogens, such as species Plesimonas shigelloides and Pseudomonas alcaligenes and genus Flavobacterium decreased significantly. In contrast, the abundance of some beneficial bacteria that can degrade pollutants and toxins (e.g., Rhodococcus erythropolis) increased significantly. Thus, the application of low concentrations (6 ~ 18 μg/l) of AVH-AgNPs in aquaculture water is relatively safe and has a positive effect on zebrafish farming.

## Introduction

Nanotechnology has provided innovative modifications to aquaculture industry, and has been used in the detection and control of aquatic pathogens, water treatment in aquaculture systems, nutrient and drug delivery, as well as DNA-nano vaccines, fish packaging, etc. (Luis et al., 2019; Fajardo et al., 2022). These meet the needs for higher quality, lower cost and green sustainability in aquaculture. Nanoparticles especially silver nanoparticles (AgNPs) have gradually been highly valued in aquaculture. AgNPs are considered an ultra-fine metallic silver element with a size of 1–100 nm or a material composed of it as a basic unit. AgNPs have been extensively studied owing to their small size, high surface-to-volume ratio, and strong surface activity. In aquaculture, AgNPs are used as antimicrobials, feed additives, and nano-vaccines (Dar et al., 2020). As feed additives, AgNPs reduced the mortality of marine shrimp (*Penaeus monodon*) infected with *Vibrio parahaemolyticus* (RathnaKumari et al., 2018) and enhanced the resistance of white shrimp (*Litopenaeus vannamei*) to white spot syndrome virus (Romo-Quiñonez et al., 2020). AgNPs feed supplements improved the ability of striped snakehead (*Channa striatus*) to resist heat and lead (Pb) and reduced the stress response of cells (Kumar et al., 2018b). In addition, the positive effects of AgNPs on intestinal health have been reported. The proper dietary level of chitosan silver nanocomposites has shown lucid benefits on gut immunity of zebrafish (*Danio rerio*) through increasing beneficial microbial populations, goblet cell density, villi length, and up-regulation of immunity related and mucin encoding genes (Udayangani et al., 2017).

AgNPs can be added and dispersed in water and are mainly used for purifying sewage and repairing wastewater (Badawy et al., 2021). Heavy metals and dyes, such as Pb ions, Congo red, and methyl orange in sewage, are absorbed by AgNPs, and loads of micro-organisms are reduced (Ganguly et al., 2021; Ogunfowora et al., 2021). AgNPs baths can also cure bacterial infections. A previous study showed that the rainbow trout (*Oncorhynchus mykiss*) infected with *Aeromonas salmonicida* did not show death or clinical symptoms after immersing in a 100 μg/ml AgNPs solution (Shaalan et al., 2018). The accumulation of AgNPs or high levels of AgNPs in aquaculture water can be toxic to aquatic animals (Lekamge et al., 2018; Hedayati et al., 2019). A previous study investigated the effects of AgNPs exposure in culture water to Nile tilapia (*Oreochromis niloticus*). Results revealed that 10 μg/l of AgNPs improved the growth performance and antioxidant enzyme activities, while more than 10 μg/l of AgNPs exposure could disturb physiological indicators and cause histopathological changes. Besides, the total bacterial count in water decreased with the increase of AgNPs concentration (Mabrouk et al., 2021).

Gut microbiota is widely recognized as an organ of its host and plays an important role in digestion and absorption, immunomodulation, and inhibition of pathogenic bacteria (Egerton et al., 2018). At present, the effects of AgNPs dispersed in aquaculture water on fish gut microbiota have not been reported before, while a comprehensive evaluation of the effect of AgNPs on cultured aquatic animals is a prerequisite for its application in aquaculture. In our previous study, the AVH-AgNPs with high dispersion stability and low toxicity were synthesized and showed a positive modulation of the bacterial community in aquaculture water (Zhang et al., 2022). In this study, the comprehensive evaluation of AVH-AgNPs effects on growth, antioxidation, immunity, gut health, and gut microbiota of zebrafish was carried out, which lays a theoretical foundation for the practical application of AgNPs in aquaculture.

## Materials and methods

### AVH-AgNPs preparation and characterization

Fresh abalone viscera were purchased from Xiamen Dao Zhiyuan Biotechnology Co., Ltd., (Fujian, China), and the abalone viscera hydrolysates (AVH) were obtained according to the previously reported method (Wang et al., 2015). The procedure for the preparation of AVH-AgNPs was also described in a previous work (Zhang et al., 2022). Briefly, 125 ml each of 1.3 mM AgNO3 and 0.7 mg/ml AVH was dissolved in 375 ml of deionized water, and the pH of the mixture was adjusted to 7.0. The reaction was carried out for 6 h at 100°C and the prepared AVH-AgNPs were further characterized. The shape and elemental composition of AVH-AgNPs were detected in a transmission electron microscope (TEM) (Tecnai F20, Germany) with a field emission transmission electron microscope (EDX) (FEI talosf200s, Germany) under 200KV voltages, and the average particle size was measured using “zeta-potential (ζ)” (Malvern ZS90, Malvern, United Kingdom).

### Zebrafish husbandry and AVH-AgNPs treatment

A total of 600 healthy adult zebrafish were purchased from a commercial aquarium in Xiamen, Fujian, China, and acclimated to the laboratory conditions for 2 weeks. During the acclimatization period, aquaculture water conditions were maintained as follows: water temperature of 25 ± 2°C; dissolved oxygen (DO) of 7.9 ± 0.1 mg/l with 12/12 h light/dark cycle; pH 7 ± 0.2.

Zebrafish were divided into four experimental groups in three replicates of 12 tanks (each tank volume is 80-L), each tank with 50 fish. The first group was a control without AVH-AgNPs addition (C0 group). The other 3 groups were added with AVH-AgNPs at doses of 6 (group C1), 9 (group C2), and 18 μg/l (group C3). All concentrations of AVH-AgNPs were selected based on the acute toxicity test (Zhang et al., 2022). During the 30-day experimental period, the fish were fed to satiation three times a day. The water was renewed at a rate of 1/3 every day, and AVH-AgNPs were added to the water timely to maintain the constant concentration.

### Sample collection

On the 10th and 20th days of the experiment, six fish were randomly collected from each tank (fasting for 24 h before sampling) and quickly anesthetized with 0.5 g/l eugenol. The body weight and length of the fish were measured, and the liver was isolated with sterile scissors from the abdomen of the fish, immediately frozen in liquid nitrogen, and stored at −80°C until further use for quantitative analysis of antioxidant enzyme activity and immune gene expression. On the 30th day, six fish were collected from each tank to measure their body weight and length, and livers were sampled for quantitative analysis of enzyme activity and gene expression. The intestine and gill tissues were collected from these fish samples and fixed in a 4% paraformaldehyde fixative solution for subsequent histopathological analysis. Additionally, another 12 fish were randomly sampled from each tank, and the intestinal tissues were separated. The intestinal tissues of every 6 fish were pooled into one sample, and all the samples were quickly frozen in liquid nitrogen and stored at −80°C for subsequent DNA extraction.

### Antioxidant enzyme activity and immune-related gene analysis

The activities of superoxide dismutase (SOD) and catalase (CAT), and glutathione (GSH) content were determined using commercial kits purchased from Nanjing Jiancheng Bioengineering Institute, Nanjing, Jiangsu, China. The determinations were carried out following the manufacturer’s protocols.

Total RNA was extracted from the liver of zebrafish using TransZol Up Plus RNA Kit (TransGen, Beijing, China) according to the manufacturer’s instructions. The purity and concentration of total RNA were detected using an ultra-micro spectrophotometer (Thermo NanoDrop 2000, Waltham, MA, United States). RNA was reverse transcribed into cDNA using TransScript All-in-One First-Strand cDNA Synthesis SuperMix for qPCR (TransGen, Beijing, China). The qPCR was performed with cDNA as a template by the intercalation fluorescence method to obtain the PCR melting curve. Ten microliter of the reaction mixtures consisted of 5 μl SYBR Green qPCR Master Mix (TransGen, Beijing, China), 1 μl of forward primer, 1 μl of reverse primer, and 3 μl of ddH_2_O. The relative mRNA expression levels were calculated by the 2^-ΔΔCT^ method (Schmittgen and Livak, 2008). Primer sequences for the reference gene (β actin) and immune-related genes are shown in Table 1.

**Table 1:** Sequences of primers used for qPCR in this study.

Gene name	Accession number	Primer name	Primer sequence (5′-3′)
Tumor necrosis factor-α (TNF-α)	BC124141	TNF-α-F	AGAAGGAGAGTTGCCTTTACCGCT
		TNF-α-R	AACACCCTCCATACACCCGACTTT
Interleukin 1β (IL-1β)	AY427649	IL-1β-F	ACGTCATCATCGCCCTGAACAGAA
		IL-1β-R	TGTAAGACGGCACTGAATCCACCA
Interleukin 10 (IL-10)	AY887900.1	IL- 10-F	CCCTATGGATGTCACGTCATG
		IL- 10-R	CATATCCCGCTTGAGTTCCTG
Interleukin 12 (IL-12)	AB183002.1	IL- 12-F	CTCAGGGAAACAGGATTACGG
		IL- 12-R	GATCTTCCTAAAGCTCCACTGG
β Defensin like 1 (DEFB1)	NM_001081553	DEFB1- F	TGTGCAAGTCTCAGTGGTGTTTGC
		DEFB1- R	TTTGCCACAGCCTAATGGTCCGAA
β actin	AF025305	β actin-F	AATCTTGCGGTATCCACGAGACCA
		β actin-R	TCTCCTTCTGCATCCTGTCAGCAA

### Histopathological investigation

The fixed tissue was dehydrated with increasing ethanol concentrations (from 75 to 100%), cleared with xylene, and embedded in paraffin. Four-micron thick paraffin was sliced *via* a microtome (Leica RM 2016, Shanghai, China). The sections were stained with hematoxylin and eosin (HE) and observed after imaging with an ortho-fluorescence microscope (Nikon Eclipse Cl, Tokyo, Japan).

### 16S rDNA Illumina sequencing and data analysis

Total DNA was extracted from the intestines with the TruSeqTM DNA Sample Prep kit (QIAGEN, Hilden, Germany) according to the manufacturer’s instructions. The qualified DNAs were sent to Shanghai Majorbio Bio-pharm Technology Co., Ltd., China, for Illumina Miseq sequencing. The single long reads and original libraries were spliced by merging paired-end sequences through the Flash (v.1.2.11) program. Sequence quality was controlled and filtered by Uparse (v.7.0.1090). The reads were optimized, and operational taxonomic units (OTUs) were classified by clustering with a 97% similarity threshold using Usearch (v.7.0) and compared the database Sliva (Release132^1^) to count the community species composition of each sample. The alpha diversity was performed using Mothur (v.1.30.2). To characterize the differences in bacterial community structure among different samples and groups at phylum, genus, and species levels, principal component analysis (PCA) and Heatmaps were performed by R project ggpolt2 package (v.2.2.1) (Gonzalez-Penagos et al., 2020).

### Statistical analysis

All data were analyzed by one-way analysis of variance (ANOVA) and verified by Duncan SPSS v.18 (Chicago, IL, United States). The differences were considered significant at *p* < 0.05 (Duncan, 1955).

## Results

### Characterization of AVH-AgNPs

**Figure 1:**
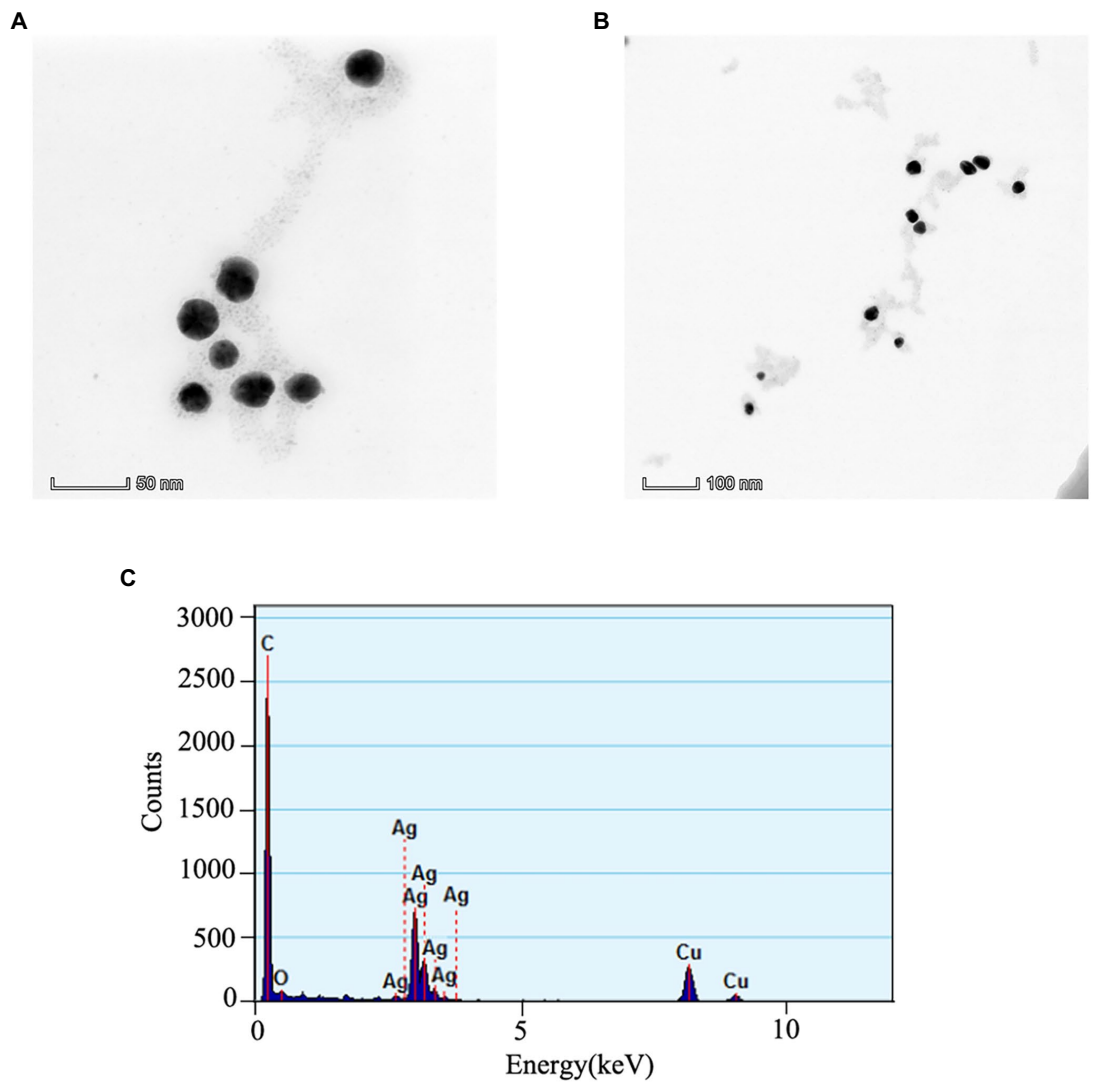
The TEM images of AVH-AgNPs at 50 nm **(A)**, 100 nm **(B)** ruler magnification and EDX spectrum acquired at 200 kV **(C)**.

The TEM images of AVH-AgNPs at 50 nm and 100 nm magnification are shown in Figures 1A,B. It can be seen that AVH-AgNPs were spherical and well-dispersed with diameter ranging from 45 nm to 70 nm. The EDX results showed that the main elements present in AVH-AgNPs were C, O, Ag and Cu. A typical absorption peak of silver element appeared at AgLα = 2.98 keV and the percentage of this element in the sample was the highest, indicating that Ag was the main constituent of this sample. The copper (Cu) element was mainly from the copper mesh used for the TEM test, and C and O elements were important constituents in the AVH (Figure 1C). Other characterization (UV–Vis, XRD, zeta potential, silver content and stability) has also been performed (Zhang et al., 2022).

### Effects of AVH-AgNPs on growth performance

**Figure 2:**
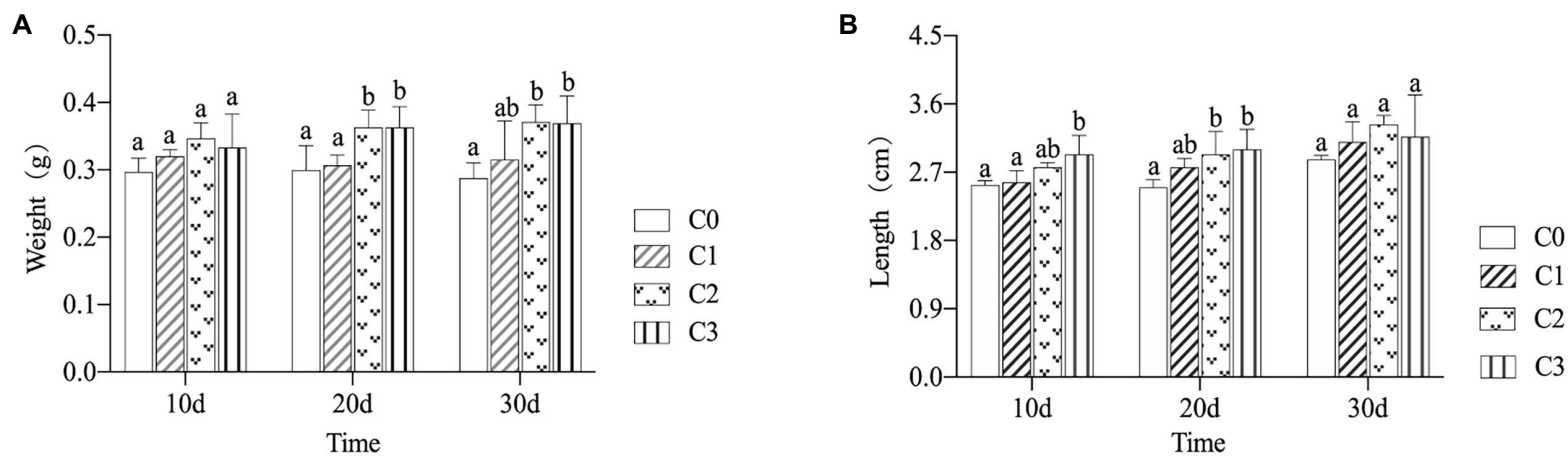
Effects of AVH-AgNPs on body weight **(A)** and length **(B)** of zebrafish. C0, C1, C2, and C3 refer to 0, 6, 9, and 18 μg/l of AVH-AgNPs, respectively. Values are expressed as mean ± SE (*n* = 3). Bars with different letters indicated significant differences (*p* < 0.05).

During the experimental period, the survival rate of zebrafish in each tank was 100%. The effects of AVH-AgNPs on the growth performance of zebrafish are shown in Figure 2. After 10 days of treatment, no significant difference in fish body weight was found among the treatment groups. With the extension of treatment time (on days 20 and 30), the fish weights in groups C2 and C3 increased significantly compared with the control group (*p* < 0.05) (Figure 2A). All the fish body lengths of treatment groups were higher than those of control group, especially in groups C3 and C2, their body lengths increased significantly on days 10 and 20 (*p* < 0.05), respectively. After 30 days of AVH-AgNPs treatment, no significant difference in body length was found among the four experimental groups (*p* > 0.05) (Figure 2B).

### Effects of AVH-AgNPs on antioxidant enzymes activities

**Figure 3:**
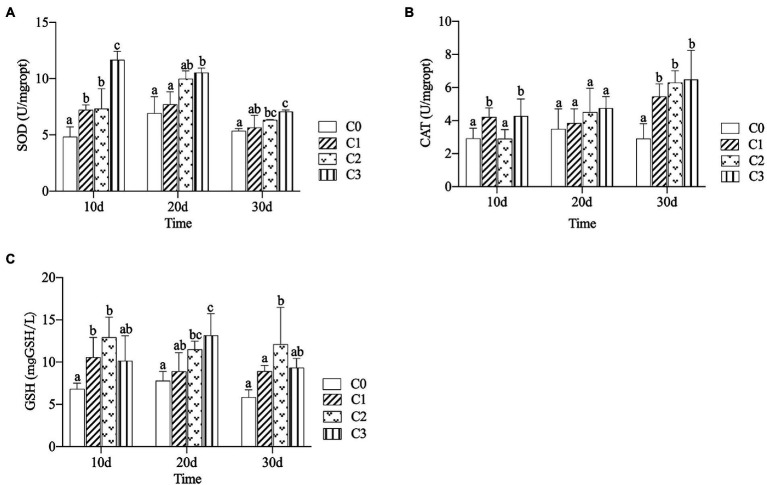
Effects of AVH-AgNPs on SOD **(A)**, CAT **(B)** and GSH content **(C)**. C0, C1, C2 and C3 refer to 0, 6, 9, and 18 μg/l of AVH- AgNPs, respectively. Values are expressed as mean ± SE (*n* = 3). Bars with different letters indicate significant differences (*p* < 0.05).

The SOD and CAT activities and GSH content in zebrafish livers are shown in Figure 3. The SOD activities increased in all AVH-AgNPs treatments throughout the whole experiment, and with the increase of AVH-AgNPs concentration, the difference between treatment and control groups reached significant levels (*p* < 0.05) (Figure 3A). AVH-AgNPs treatments showed inductive effects on CAT activity, and all the activities in treatment groups were higher than those in controls, but different treatments showed different effects. On day 10, CAT activities of groups C1 and C3 were significantly higher than those of the control group, while no significant difference was found in CAT activity between C2 and C0. On day 20, the differences among the experimental groups did not reach a significant level. On day 30, all the treatment groups were significantly higher than the control group (*p* < 0.05), and the higher concentration treatment had a higher CAT activity (Figure 3B). All the treatments increased the GSH content to different degrees. At the initial stage of the experiment, the difference between the treatments and controls was more obvious. With time, the differences were not so obvious. On day 30, only the C2 group was significantly higher than the control group (Figure 3C).

### Effect of AVH-AgNPs on immune gene expression

**Figure 4:**
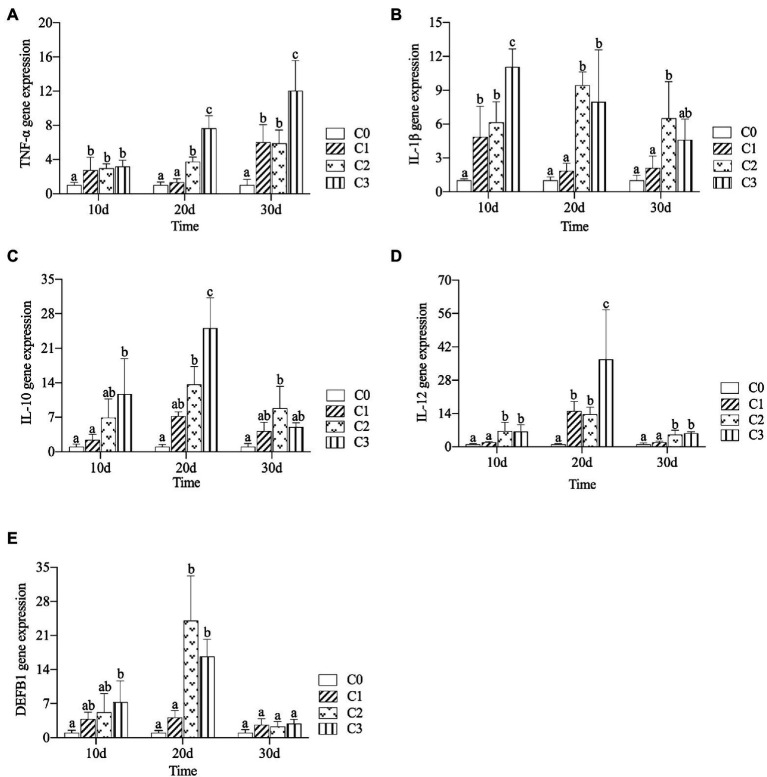
Effects of AVH-AgNPs on the gene expression levels of TNF-α **(A)**, IL-1β **(B)**, IL-10 **(C)**, IL-12 **(D)**, and DEFB1 **(E)**. C0, C1, C2, and C3 refer to 0, 6, 9, and 18 μg/l of AVH-AgNPs, respectively. Values are expressed as mean ± SE (*n* = 3). Bars with different letters indicate significant differences (*p* < 0.05).

The effects of AVH-AgNPs on gene expression levels of TNF-α, IL-1β, IL-10, IL-12, and DEFB1 are shown in Figure 4. Compared with the control group, all the gene expression levels of TNF-α in treatments were upregulated, and the higher concentration and longer treatment time had the more obvious upregulation effect (Figure 4A). The effect trends of AVH-AgNPs on the gene expression levels of IL-1β, IL-10, IL-12, and DEFB1 were similar (Figures 4B–E). All of these genes were upregulated to varying degrees after AVH-AgNPs treatment, in which the upregulation effect on IL-1β was the most obvious. The upregulation effects of the C2 and C3 groups were more obvious than those of the C1 group. The upregulation effect was most obvious on the 20th day (IL-1β was also significantly upregulated on the 10th day). However, the upregulation effects of these genes decreased with the extension of the experimental time. On day 30, the expression levels of the DEFB1 gene in the treatment groups were not significantly different from that of the control.

### Measurement and analysis of intestinal structure

**Figure 5:**
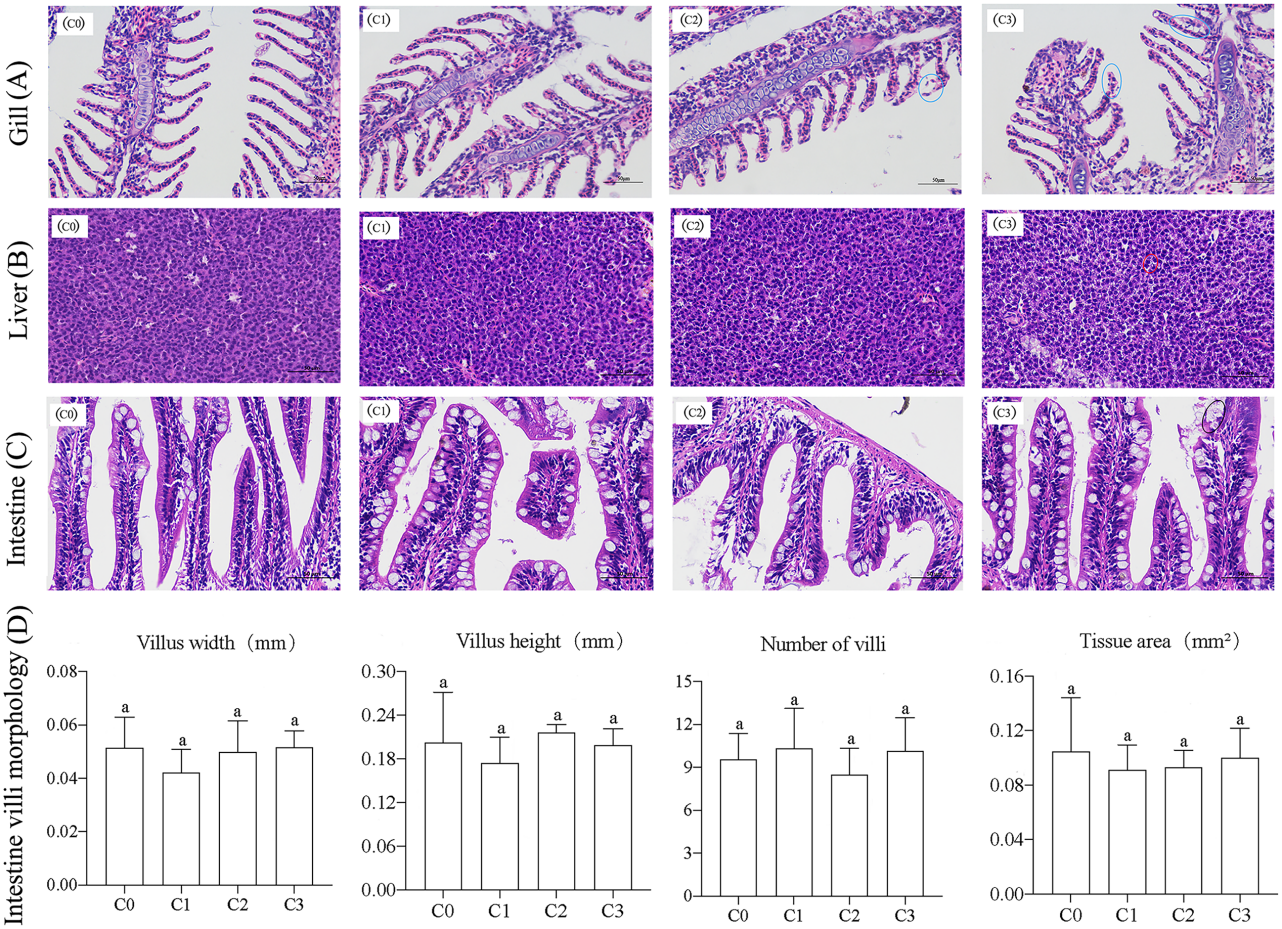
Effects of AVH-AgNPs on gills **(A)**, livers **(B)**, intestines **(C)**, and intestinal villi **(D)** of zebrafish. C0, C1, C2, and C3 refer to 0, 6, 9, and 18 μg/l of AVH-AgNPs, respectively. Values are expressed as mean ± SE (*n* = 3). Bars with the same letters indicate no significant differences (*p* > 0.05).

The histological analysis of zebrafish gills, liver, and intestines are showed in Figure 5. All groups had normal, healthy gills without obvious lesions, except that in groups C2 and C3, there was a phenomenon of sub-epithelium cellular infiltrations, and a slight loss of secondary lamella of epithelial cells (marked with blue circle in Figure 5A). Hepatic tissues in all groups showed a regular and normal morphological structure. Hepatocytes in C3 group had mild granular degeneration, but there was no obvious abnormality in morphological structure and inflammatory cellular infiltrations (Figure 5B). The histological examination of intestinal sections from C3 group showed few intestinal chorionic mucosal epithelial cells shed (marked with black circle in Figure 5C). However, the intestinal mucosal epithelium of other groups was intact and no cell exfoliation was observed. Also, statistical analysis showed that, compared with the control group, there were no significant changes in the number, height, width of villi and intestinal tissue area of all AVH-AgNPs treatment groups (Figure 5D).

### Effects of AVH-AgNPs on gut microbiota

#### Alpha diversity

After Illumina sequencing, a total of 563,537 effective sequences were obtained from 16 samples, with an average sequence length of 414.75 ± 1.33 bp. Each sample had 40,222 ~ 62,918 valid sequences, and the coverages of all samples were greater than 0.99. To avoid the analysis error caused by different sequencing depths, 40,000 sequences were normalized from each sample for subsequent analysis.

**Figure 6:**
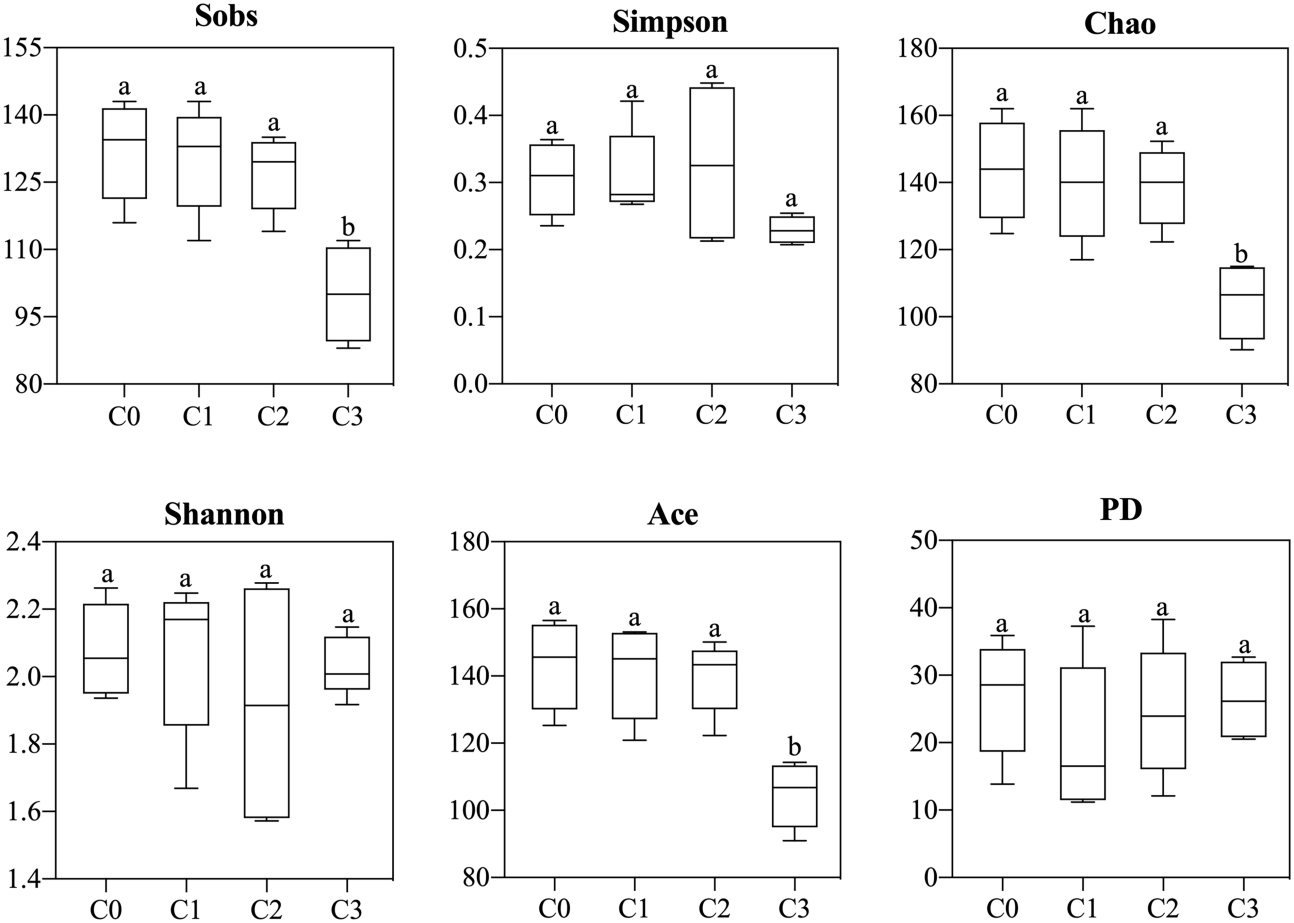
Effects of AVH-AgNPs on alpha-diversity indices of gut microbes of zebrafish. C0, C1, C2, and C3 refer to 0, 6, 9, and 18 μg/l of AVH-AgNPs, respectively. Values are expressed as mean ± SE (*n* = 3). Bars with different letters indicate significant differences (*p* < 0.05).

AVH-AgNPs treatment had a certain effect on the alpha diversity of zebrafish gut microbiota, and different treatments had different effects. As shown in Figure 6, compared with the control group, the Sobs, Chao, and ACE indices in the C3 group decreased significantly, while no significant changes were found in groups C1 and C2 and other diversity indices in treatment groups did not change significantly.

#### Principal component analysis

**Figure 7:**
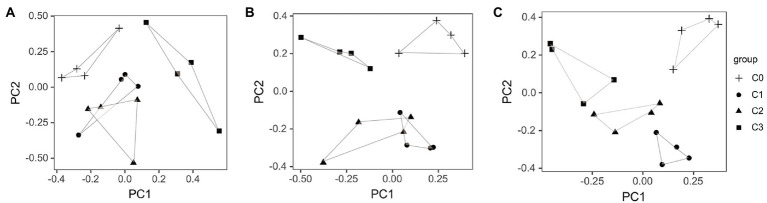
Principal component analysis (PCA) of zebrafish gut microbiota at phylum **(A)**, genus **(B)**, and species **(C)** levels. C0, C1, C2, and C3 refer to 0, 6, 9, and 18 μg/l of AVH-AgNPs, respectively.

PCA showed that AVH-AgNPs affected the intestine bacterial community structure at phylum, genus, and species levels, and the effects were different in different treatments (Figure 7). All the control samples were separated from the AVH-AgNPs treatments. Although the samples of C1 and C2 overlapped at phylum and genus levels, all the other samples from different groups were separated from each other and clustered separately at all taxon levels. Groups C1 and C2 were closer, while group C3 was farther apart from other groups, indicating that the effects of AVH-AgNPs on the intestine microbial community structure of zebrafish showed a concentration-dependent trend from phylum to species levels. The higher concentration of AVH-AgNPs, the more obvious the effect.

#### Analysis of intestine microbial composition

**Figure 8:**
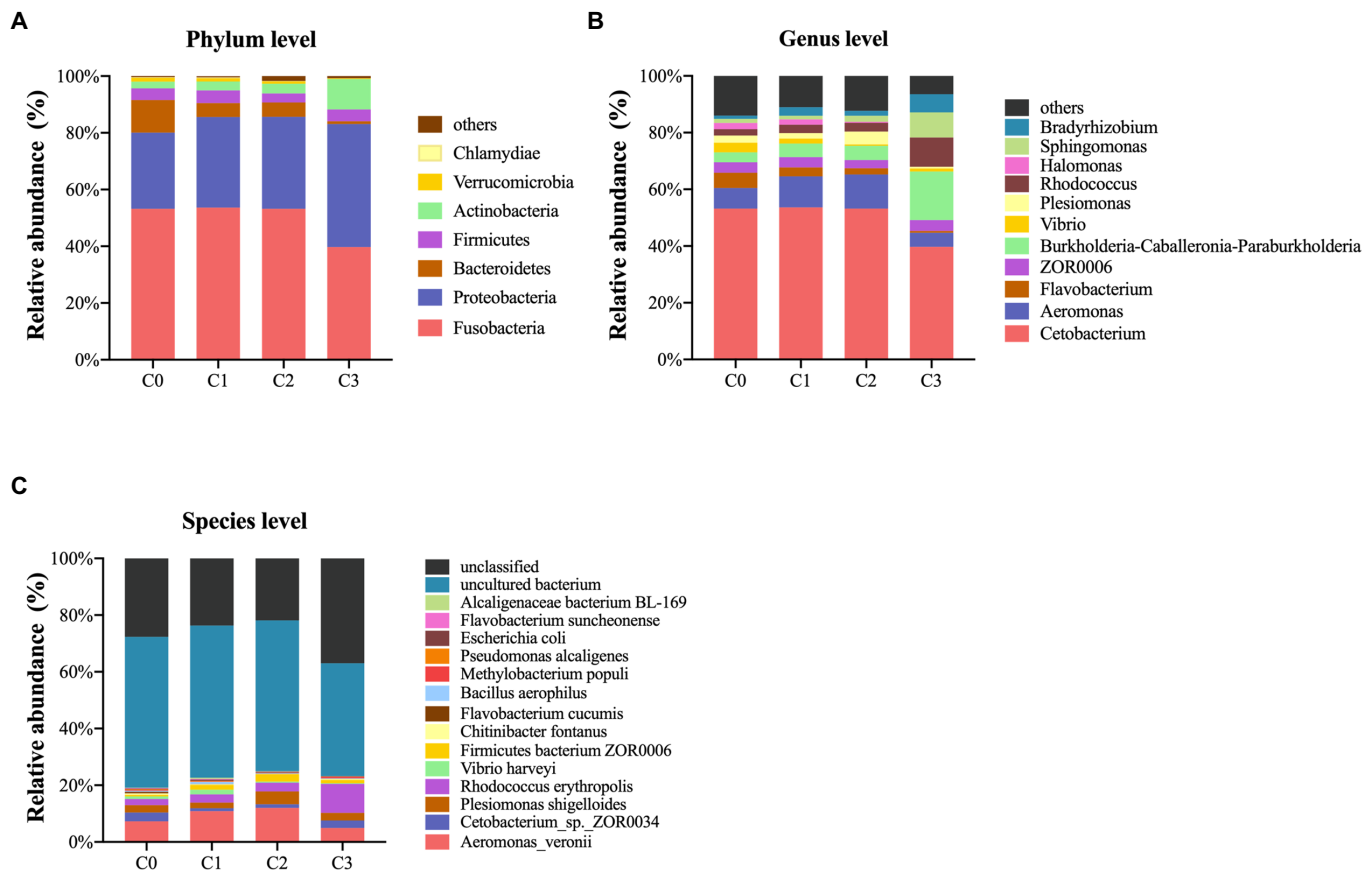
Gut microbial composition of zebrafish at phylum **(A)**, genus **(B)** and species **(C)** levels. C0, C1, C2, and C3 indicate AVH-AgNPs treatments with concentrations of 0, 6, 9, and 18 μg/l, respectively.

The gut microbial composition at phylum, genus, and species levels are shown in Figure 8. A total of 19 phyla were detected in zebrafish guts, and the most dominant phylum was Fusobacteria (occupied 53.18%), followed by Proteobacteria (26.86%), Bacteroidetes (11.51%), Firmicutes (4.14%), Actinobacteria (2.35%) and Verrucomicrobia (1.40%). Chlamydiae (0.14%) was also detected, and others (< 0.1%) were less detected (Figure 8A).

A total of 238 genera were detected, and the abundances of 38 of the top 50 genera were identified by comparison (the others were “unclassified” or “norank”). The most dominant genus was *Cetobacterium* (53.18%), followed by *Aeromonas* (7.29%) and *Flavobacterium* (5.4%). The genera with abundances ranged from 1 to 4%, in order of high to low, which were as follows: *ZOR0006*, *Burkholderia-Caballeronia-Paraburkholderia, Vibrio*, *Plesiomonas*, *Rhodococcus*, *Halomonas*, *Sphingomonas* and *Bradyrhizobium*, and others were less than 1% (Figure 8B).

A total of 319 species were detected, of which 74 species were identified, and the dominant species (arranged from high to low in abundance) were: *Aeromonas veronii*, *Cetobacterium* sp. *ZOR0034*, *Plesimonas shigelloides,* and *Rhodococcus erythropolis* (7.28 to 2.22%). Other abundant species were *Vibrio harveyi*, *Firmicutes bacterium ZOR0006*, *Chitinibacter fontanus,* and *Flavobacterium cucumis* (accounting for 0.87 to 0.5%). Besides, *Bacillus aerophilus*, *Methylobacterium populi*, *Pseudomonas alcaligenes*, *Escherichia coli*, *Flavobacterium suncheonense*, *Alcaligenaceae bacterium BL-169,* etc., were also detected, and their abundances were more than 0.1% (Figure 8C).

#### Effects of AVH-AgNPs on gut microbiota

**Figure 9:**
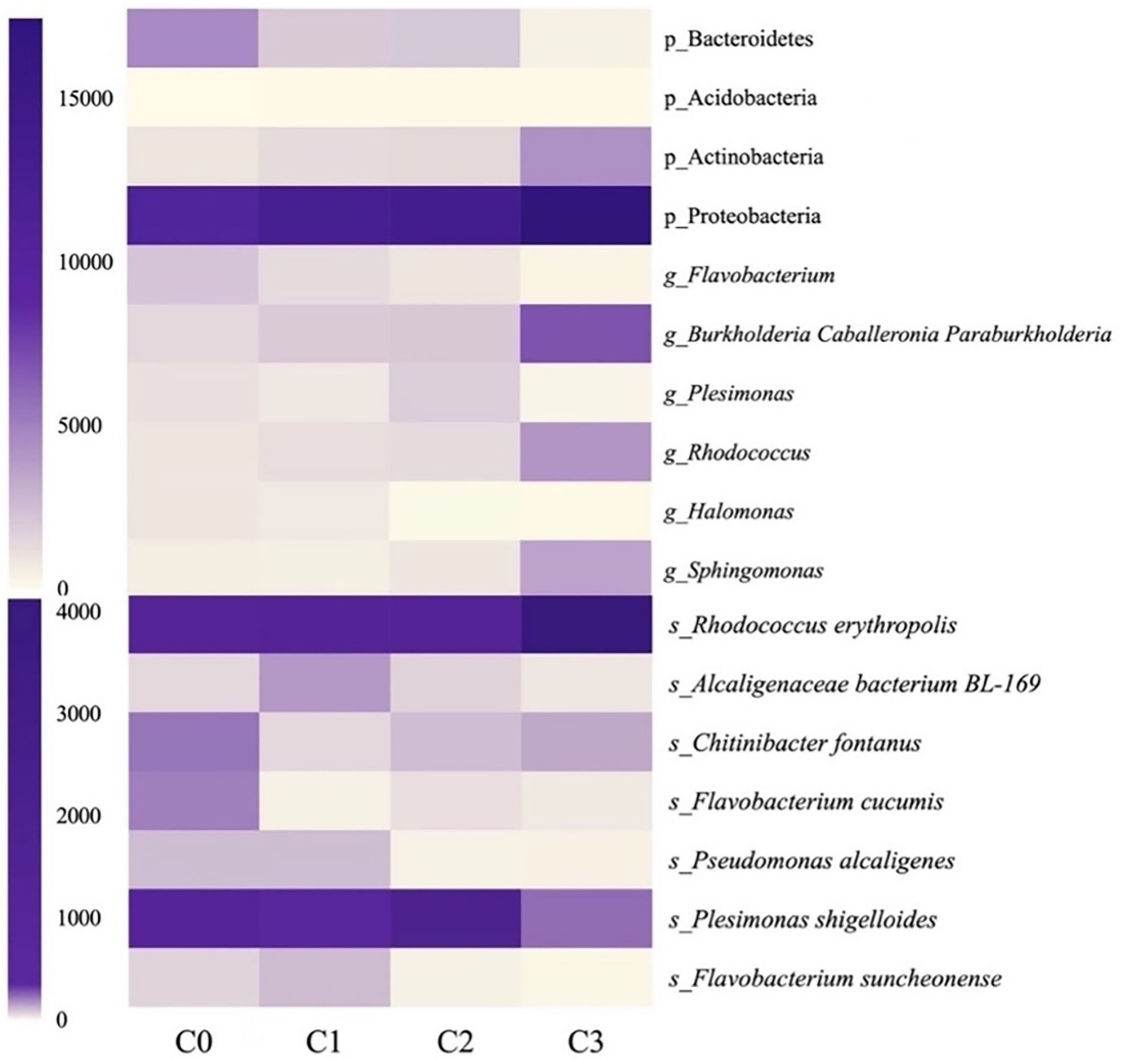
Heatmap analysis of the identified bacteria with significant changes after AVH-AgNPs treatment. C0, C1, C2, and C3 indicate AVH-AgNPs treatments with concentrations of 0, 6, 9, and 18 μg/l, respectively. P_, g_, and s_ represent classifications at phylum, genus, and species levels, respectively.

Figure 9 shows the identified bacteria with significant changes in abundance at phylum, genus, and species levels after AVH-AgNPs treatment. At the phylum level, the abundance of Proteobacteria and Actinobacteria in all three treatments increased, especially in the C3 group, and the increase reached a significant level compared to the control group. The abundance of Acidobacteria in the C2 and C3 groups were significantly higher than those in the control group, while the abundance of Bacteroidetes decreased significantly in all three treatments.

At the genus level, in the identified genera with an abundance of more than 1%, *Halomonas* and *Flavobacterium* decreased significantly in all three AVH-AgNPs treatments. *Plesimonas* decreased significantly in C2 and C3 groups, while *Burkholderia-Caballeronia-Paraburkholderia*, *Sphingomonas,* and *Rhodococcus* increased significantly in the C3 group compared with the control group.

At the species level, in the identified species with an abundance of more than 0.1%, *F. cucumis* decreased significantly in all three treatments, *P. shigelloides*, *P. alcaligenes*, and *F. suncheonense* decreased significantly in C2 and C3 groups, and *C. fontanus* decreased significantly in C1 and C2 groups. *Alcaligenaceae bacterium BL-169* increased significantly in the C1 group, and *R. erythropolis* increased significantly in the C3 group.

## Discussion

The synthesis of AgNPs mainly includes physical, chemical and biological routes. AgNPs prepared by physiochemical methods are easy to aggregate, and the chemical reagents used to stabilize the nanoparticles (such as surfactants, stabilizers, etc.) are harmful and easy to cause environmental pollution (Hasan et al., 2020). Compared with physiochemical methods, biosynthesis methods have advantages such as low cost, eco-friendly and easy availability (Olga et al., 2022). Many biological substances such as polysaccharides, polyphenols, proteins and extracts from plants or animal tissues are considered suitable capping agents and stabilizers (Sivaramasamy et al., 2016; Kharissova et al., 2019; Jian et al., 2020; Dhar et al., 2021). The AVH-AgNPs used in the present study had been biosynthesized using abalone viscera hydrolysates obtained through primary enzymatic hydrolysis, and showed low toxicity, high dispersion stability and antimicrobial properties (Zhang et al., 2022).

SOD and CAT are the most important antioxidant enzymes for scavenging and repairing reactive oxygen species (ROS). Previous studies showed that dietary supplementation with AgNPs significantly improved the growth of poultry (Mahmoud, 2012), weaned pigs (Fondevila et al., 2009), and striped snakehead and the activities of SOD and GST of striped snakehead (Kumar et al., 2018a). Oral administration of 42.599 mg/kg (equivalent to 1/10 lethal concentration, LC50) AgNPs increased the blood GSH content of irradiated mice and reduced the liver DNA damage caused by the irradiation (Amin et al., 2016). Oral supplementation of AgNPs in the Drinking Water of chickens at low doses (2 ppm) enhanced the antioxidant enzymes and improved growth (Fouda et al., 2021). Immune gene expression is one of the effective ways to evaluate the immune response of animals. Dietary supplementation with 20 g/kg chitosan silver nanocomposites increased the expression of TNF-α, IL-10, IL-12, and DEFB1 genes and enhanced the intestinal immunity of zebrafish (Udayangani et al., 2017). Similarly, adding 50 μg/l AgNPs in water induced the expression of TNF-α and IL-1β genes of skin-wounded zebrafish within 24 h (Seo et al., 2017).

In the present study, low concentrations of AVH-AgNPs (6 to 18 μg/l, equivalent to 1/30 to 1/10 of the LC50) (Zhang et al., 2022) were added to the culture water of zebrafish for 30 days. The higher growth performance, SOD and CAT activities, and GSH contents of zebrafish were found, which indicated that AVH-AgNPs improved the antioxidant capacity of zebrafish. The expression levels of immune genes (TNF-α, IL-12, IL-12, IL-10, and DEFB1) were also significantly upregulated. In addition, the histopathological analysis showed that AVH-AgNPs did not cause significant damage to the tissue structure of gills, livers, and intestines of zebrafish within the experimental concentration range. These results indicate that low concentrations of AVH-AgNPs supplemented in aquaculture water are safe and have antioxidant and immunomodulatory effects on aquatic animals.

After AVH-AgNPs treatment, the abundance of some bacteria associated with disease decreased significantly. *P. shigelloides* have been reported as a zoonotic pathogen of gastrointestinal diseases and are responsible for the high mortality of many farmed fish (Behera et al., 2018; Yilmaz, 2019; Cortés-Sánchez et al., 2021). It is abundance in control was as high as 2.55% but significantly decreased to 0.27 and 0.58% in the C2 and C3 groups, respectively. *P. alcaligenes* is considered to be a conditional pathogen for humans and animals (Bowman, 2005) and could be isolated from diseased Asian Swamp Eel (*Monopterus albus*) (Ma and Shu, 2000), Chinese Sturgeon (*Acipenser sinensis*) (Xu et al., 2015), Large yellow croaker (*Pseudosciaena crocea*) (Huang et al., 2019) and Koi carp (*Cyprinus rubrofuscus*) (Bai et al., 2021). *P. alcaligenes* is a serious sub-health problem in aquaculture (Suzuki et al., 2013). The abundance of this bacterium was 0.25% in the control group but decreased significantly to 0.032 and 0.037% in the C2 and C3 groups, respectively.

Compared with the control group, the abundance of *F. cucumis* decreased significantly in all three treatments, and *F. suncheonense* decreased significantly in C2 and C3 groups. Both strains were isolated from greenhouse soil in Korea and had polysaccharide decomposition potential (Weon et al., 2007; Tashkandy et al., 2016). *F. cucumis* was isolated from diseased fish (Genbank accession number: KU851954), suggesting a link to fish disease. In addition, there are many unknown species of *Flavobacterium* decreased significantly. *Flavobacterium* contains many aquatic pathogens and is one of the pathogenic genera of zebrafish (Gonzalez-Penagos et al., 2020). In some areas, flavobacteriosis causes more fish deaths in both wild and farmed fish than all other pathogens in a combination. In addition to the commonly known pathogens, such as *Flavobacterium columnar*, *Flavobacterium branchiarum*, and *Flavobacterium psychrophilum*, many unidentified *Flavobacterium* species cause flavobacteriosis (LaFrentz et al., 2021).

*Halomonas* decreased significantly in all treatment groups. This genus mainly presents in saline environments, and its pathogenicity to humans, animals, and algae has been described (Kim et al., 2013). Additionally, the abundance of some potential toxin-degrading bacteria, such as *Rhodococcus* and *R. erythropolis*, increased significantly after AVH-AgNPs treatment. The *R. erythropolis* could degrade organic pollutants (Bai et al., 2020) and mycotoxins, reducing the embryonic mortality of zebrafish caused by mycotoxins (Garai et al., 2021). After AVH-AgNPs treatment, the abundance of bacterial taxa mentioned above changed significantly, which played important roles in controlling environmental pathogens and reducing the incidence of disease in farmed animals.

After AVH-AgNPs treatment, some functional bacteria that help degrade acephate and polycyclic aromatic hydrocarbons and other pollutants, such as *Alcaligenaceae bacterium BL-169*, *Burkholderia-Caballeronia-Paraburkholderia,* and *Sphingomonas* (Felföldi et al., 2010; Lin et al., 2022), increased significantly in C1 and C3 groups, respectively, which would help farmed animals cope with environmental pollution and improve stress resistance. In addition, the abundance of *C. fontanus* decreased significantly in some treatment groups, and its role needs further study.

In previous studies, AgNPs were usually used as feed additives in aquaculture, while in this study, the AVH-AgNPs can be stably dispersed in aquaculture water and have been proved to exert positive bioeffects on zebrafish regarding the growth performance, immune gene expression and gut microbiota modulation. Compared with the AgNPs supplement feeds, the application of dispersing AVH-AgNPs in aquaculture water is more convenient.

## Conclusion

Low concentrations of AVH-AgNPs (6 ~ 18 μg/l) dispersed in aquaculture water did not cause significant damage to the morphology of gills, livers, and intestinal tissues of zebrafish and promoted the growth, induced the increase of SOD and CAT activities and GSH content, and upregulated the immune-related gene expression of zebrafish. After AVH-AgNPs treatment, some pathogenic or opportunistic pathogens significantly decreased in zebrafish intestines, while the beneficial bacteria that could degrade pollutants and improve the intestinal environment increased significantly. Therefore, applying a low concentration of AVH-AgNPs in aquaculture water is relatively safe and has positive significance in improving antioxidation and immunity, controlling intestinal pathogens, and reducing the incidence of cultured animals. Considering it might be more convenient to apply in water than in feed, we suggest that AVH-AgNPs have good application prospects in aquaculture.

## Data availability statement

The original contributions presented in the study are included in the article/supplementary material, further inquiries can be directed to the corresponding authors.

## Ethics statement

The animal study was reviewed and approved by the Animal Ethics Committee of Jimei University (Acceptance no. JMULAC201159).

## Author contributions

JN and ZY conducted the experiments and wrote the manuscript. YZ performed the statistical analysis. YM, HX, and WJ contributed to the conception and designed of the study. All authors contributed to manuscript revision, read, and approved the submitted version.

## Funding

This work was supported by Natural Science Foundation of Fujian Province (grant no: 2020 J01662), Xiamen Science and Technology Planning Project (grant no: 2022CXY0307) and the Program of Institute of Respiratory Diseases, Xiamen Medical College (grant no: HXJB-14), China.

## Conflict of interest

The authors declare that they have no known competing financial interests or personal relationships that could have appeared to influence the work reported in this paper.

## Publisher’s note

All claims expressed in this article are solely those of the authors and do not necessarily represent those of their affiliated organizations, or those of the publisher, the editors and the reviewers. Any product that may be evaluated in this article, or claim that may be made by its manufacturer, is not guaranteed or endorsed by the publisher.

## Footnotes

^1^ http://www.arb-silva.de

## References

[ref1] Amin Y. M. Hawas A. M. El-Batal A. Hassan S. H. Elsayed M. E. (2016). Subchronic effect of silver nanoparticles following 28 days of repeated Oral administration on oxidative stress, inflammatory biomarkers and DNA fragmentation in Normal and irradiated rats. Br. J. Pharmacol. Toxicol. 7, 36–50. doi: 10.19026/bjpt.7.3337

[ref2] Badawy A. A. Ghanem A. F. Yassin M. A. Youssef A. M. Rehim M. H. A. (2021). Utilization and characterization of cellulose nanocrystals decorated with silver and zinc oxide nanoparticles for removal of lead ion from wastewater. Environ. Nanotechnol. Monitor. Manage. 16:100501. doi: 10.1016/j.enmm.2021.100501

[ref3] Bai J. Y. Huo X. Hu A. L. Sun J. (2021). Characterization of pathogenic *Pseudomonas alcaligenes* isolated from koi carp in China. J. Aquat. Anim. Health 33, 243–251. doi: 10.1002/aah.10141 34327768

[ref4] Bai F. Tian H. Ma J. (2020). Landfill leachate treatment through the combination of genetically engineered bacteria *Rhodococcus erythropolis* expressing *Nirs* and *AMO* and membrane filtration processes. Environ. Pollut. 263:114061. doi: 10.1016/j.envpol.2020.114061 32268229

[ref5] Behera B. K. Bera A. K. Paria P. Das A. Parida P. K. Kumari S.. (2018). Identification and pathogenicity of *Plesiomonas shigelloides* in silver carp. Aquaculture 493, 314–318. doi: 10.1016/j.aquaculture.2018.04.063

[ref6] Bowman J. P. (2005). “Genus: pseudomonas,” in Bergey’s Manual of Systematic Bacteriology. eds. Brenner D. J. Krieg N. R. Staley J. R. (New York: Springer), *Vol 2*, 323–379.

[ref7] Cortés-Sánchez A. D. J. Espinosa-Chaurand L. D. Díaz-Ramirez M. Torres-Ochoa E. (2021). *Plesiomonas*: a review on food safety, fish-borne diseases, and tilapia. Sci. World J. 2021:3119958. doi: 10.1155/2021/3119958 PMC8478591 34594160

[ref8] Dar A. H. Rashid N. Majid I. Hussain S. Dar M. A. (2020). Nanotechnology interventions in aquaculture and seafood preservation. Crit. Rev. Food Sci. Nutr. 60, 1912–1921. doi: 10.1080/10408398.2019.1617232 31131615

[ref9] Dhar S. A. Chowdhury R. A. Das S. Nahian M. K. Islam D. Gafur M. A. (2021). Plant-mediated green synthesis and characterization of silver nanoparticles using Phyllanthus emblica fruit extract. Material. Today Proc. 42, 1867–1871. doi: 10.1016/j.matpr.2020.12.222

[ref10] Duncan D. B. (1955). Multiple range and multiple F tests. Biometrics 11, 1–42.

[ref11] Egerton S. Culloty S. Whooley J. Stanton C. Ross R. P. (2018). The gut microbiota of marine fish. Front. Microbiol. 9:873. doi: 10.3389/fmicb.2018.00873 29780377 PMC5946678

[ref12] Fajardo C. Martinez-Rodriguez G. Blasco J. Mancera J. M. Thomas B. De Donato M. (2022). Nanotechnology in aquaculture: applications, perspectives and regulatory challenges. Aquacult. Fish. 7, 185–200. doi: 10.1016/j.aaf.2021.12.006

[ref13] Felföldi T. Székely A. J. Gorál R. Barkács K. Scheirich G. András J.. (2010). Polyphasic bacterial community analysis of an aerobic activated sludge removing phenols and thiocyanate from coke plant effluent. Bioresour. Technol. 101, 3406–3414. doi: 10.1016/j.biortech.2009.12.053 20093025

[ref14] Fondevila M. Herrer R. Casallas M. C. Abecia L. Ducha J. J. (2009). Silver nanoparticles as a potential antimicrobial additive for weaned pigs. Anim. Feed Sci. Technol. 150, 259–269. doi: 10.1016/j.anifeedsci.2008.09.003

[ref001] Fouda M. Dosoky W. Radwan N. Abdelsalam N. Taha A. Khafaga A. (2021). Oral administration of silver nanoparticles-adorned starch as a growth promotor in poultry: immunological and histopathological study. Int. J. Biol. Macromol. 187, 830–839. doi: 10.1016/j.ijbiomac.2021.07.157 34331979

[ref15] Ganguly K. Dutta S. D. Patel D. K. Lim K.-T. (2021). Silver nanoparticles for wastewater treatment. Aquananotechnology 2021, 385–401. doi: 10.1016/B978-0-12-821141-0.00016-1

[ref16] Garai E. Risa A. Varga E. Cserháti M. Kriszt B. Urbányi B.. (2021). Evaluation of the multimycotoxin-degrading efficiency of *Rhodococcus erythropolis* NI1 strain with the three-step zebrafish microinjection method. Int. J. Mol. Sci. 22:724. doi: 10.3390/ijms22020724 33450918 PMC7828439

[ref17] Gonzalez-Penagos C. E. Zamora-Briseno J. A. Cerqueda-Garcia D. Amendola-Pimenta M. Perez-Vega J. A.. (2020). Alterations in the gut microbiota of zebrafish (*Danio rerio*) in response to water-soluble crude oil components and its mixture with a chemical dispersant. Front. Public Health 8:584953. doi: 10.3389/fpubh.2020.584953 33194990 PMC7649143

[ref18] Hasan M. Rafique S. Zafar A. Loomba S. Khan R. Hassan S. G.. (2020). Physiological and anti-oxidative response of biologically and chemically synthesized iron oxide: Zea mays a case study. Heliyon 6:e04595. doi: 10.1016/j.heliyon.2020.e04595 32923707 PMC7475124

[ref19] Hedayati S. A. Farsani H. G. Naserabad S. S. Hoseinifar S. H. Van Doan H. (2019). Protective effect of dietary vitamin E on immunological and biochemical induction through silver nanoparticles (AgNPs) inclusion in diet and silver salt (AgNO3) exposure on zebrafish (*Danio rerio*). Comp. Biochem. Physiol. 222, 100–107. doi: 10.1016/j.cbpc.2019.04.004 31004833

[ref20] Huang L. Zuo Y. Jiang Q. Su Y. Qin Y. Xu X.. (2019). A metabolomic investigation into the temperature-dependent virulence of *pseudomonas plecoglossicida* from large yellow croaker (*Pseudosciaena crocea*). J. Fish Dis. 42, 431–446. doi: 10.1111/jfd.12957 30659613

[ref21] Jian W. Ma Y. Zhu X. Zhang N. Lin L. Jia B.. (2020). Quantitative insight into dispersity and antibactericidal capability of silver nanoparticles noncovalently conjugated by polysaccharide-protein complexes. Int. J. Biol. Macromol. 150, 459–467. doi: 10.1016/j.ijbiomac.2020.02.098 32057866

[ref22] Kharissova O. V. Kharisov B. I. Oliva Gonzalez C. M. Mendez Y. P. Lopez I. (2019). Greener synthesis of chemical compounds and materials. R. Soc. Open Sci. 6:191378. doi: 10.1098/rsos.191378 31827868 PMC6894553

[ref23] Kim K. K. Lee J.-S. Stevens D. A. (2013). Microbiology and epidemiology of *Halomonas* species. Future Microbiol. 8, 1559–1573. doi: 10.2217/fmb.13.108 24266356

[ref24] Kumar N. Krishnani K. K. Gupta S. K. Singh N. P. (2018b). Effects of silver nanoparticles on stress biomarkers of *Channa striatus*: immuno-protective or toxic? Environ. Sci. Pollut. Res. 25, 14813–14826. doi: 10.1007/s11356-018-1628-8 29541984

[ref25] Kumar N. Krishnani K. Kumar P. Sharma R. Baitha R. Singh D. K.. (2018a). Dietary nano-silver: does support or discourage thermal tolerance and biochemical status in air-breathing fish reared under multiple stressors? J. Therm. Biol. 77, 111–121. 30196889 10.1016/j.jtherbio.2018.08.011

[ref26] LaFrentz B. R. Kralova S. Burbick C. R. Alexander T. L. Phillips C. W. Griffin M. J.. (2021). The fish pathogen *Flavobacterium columnare* represents four distinct species: *Flavobacterium columnare*, *Flavobacterium covae* sp. nov., *Flavobacterium davisii* sp. nov. and *Flavobacterium oreochromis* sp. nov., and emended description of *Flavobacterium columnare*. Syst. Appl. Microbiol. 45:126293. doi: 10.1016/j.syapm.2021.126293 35026686

[ref27] Lekamge S. Ball A. S. Shukla R. Nugegoda D. (2018). The toxicity of nanoparticles to organisms in freshwater. Rev. Environ. Contam. Toxicol. 248, 1–80. doi: 10.1007/398_2018_18 30413977

[ref28] Lin Z. Pang S. Zhou Z. Wu X. Li J. Huang Y.. (2022). Novel pathway of acephate degradation by the microbial consortium ZQ01 and its potential for environmental bioremediation. J. Hazard. Mater. 426:127841. doi: 10.1016/j.jhazmat.2021.127841 34844804

[ref29] Luis A. I. S. Campos E. V. R. de Oliveira J. L. Fraceto L. F. (2019). Trends in aquaculture sciences: from now to use of nanotechnology for disease control. Rev. Aquac. 11, 119–132. doi: 10.1111/raq.12229

[ref30] Ma Y. Z. Shu M. A. (2000). Isolation and identification of *pseudomonas Alcaligenes* in Monopterus albus. Freshwater Fish. 30, 33–34.

[ref31] Mabrouk M. M. Mansour A. T. Abdelhamid A. F. Abualnaja K. M. Mamoon A. Gado W. S.. (2021). Impact of aqueous exposure to silver nanoparticles on growth performance, redox status, non-specific immunity, and histopathological changes of Nile tilapia, *Oreochromis niloticus*, challenged with *Aeromonas hydrophila*. Aquacul. Rep. 21:100816. doi: 10.1016/j.aqrep.2021.100816

[ref32] Mahmoud U. T. (2012). Silver nanoparticles in poultry production. J. Adv. Vet. Res. 2, 303–306.

[ref33] Ogunfowora L. A. Iwuozor K. O. Ighalo J. O. Igwegbe C. A. (2021). Trends in the treatment of aquaculture effluents using nanotechnology. Clean. Mat. 2:100024. doi: 10.1016/j.clema.2021.100024

[ref34] Olga M. Jana M. Anna M. Irena K. Jan M. Alena Č. (2022). Antimicrobial properties and applications of metal nanoparticles biosynthesized by green methods. Biotechnol. Adv. 58:107905. doi: 10.1016/j.biotechadv.2022.107905 35031394

[ref35] RathnaKumari P. Kolanchinathan P. Siva D. Abirami B. Masilamani V. John G.. (2018). Antibacterial efficacy of seagrass Cymodocea serrulate-engineered silver nanoparticles against prawn pathogen Vibrio parahaemolyticus and its combative effect on the marine shrimp Penaeus monodon. Aquaculture 493, 158–164. doi: 10.1016/j.aquaculture.2018.04.061

[ref36] Romo-Quiñonez C. R. Álvarez-Sánchez A. R. Álvarez-Ruiz P. Chávez-Sánchez M. C. Bogdanchikova N. Pestryakov A.. (2020). Evaluation of a new Argovit as an antiviral agent included in feed to protect the shrimp *Litopenaeus vannamei* against white spot syndrome virus infection. PeerJ 8:e8446. doi: 10.7717/peerj.8446 32149020 PMC7049459

[ref37] Schmittgen T. D. Livak K. J. (2008). Analyzing real-time PCR data by the comparative CT method. Nat. Protoc. 3, 1101–1108. doi: 10.1038/nprot.2008.73 18546601

[ref38] Seo S. B. Dananjaya S. Nikapitiya C. Park B. K. Gooneratne R. Kim T.-Y.. (2017). Silver nanoparticles enhance wound healing in zebrafish (*Danio rerio*). Fish Shellfish Immunol. 68, 536–545. doi: 10.1016/j.fsi.2017.07.057 28757200

[ref39] Shaalan M. El-Mahdy M. Theiner S. Dinhopl N. El-Matbouli M. Saleh M. (2018). Silver nanoparticles: their role as antibacterial agent against *Aeromonas salmonicida* subsp. Salmonicida in rainbow trout (*Oncorhynchus mykiss*). Res. Vet. Sci. 119, 196–204. doi: 10.1016/j.rvsc.2018.06.019 29958154

[ref40] Sivaramasamy E. Zhiwei W. Li F. Xiang J. (2016). Enhancement of Vibriosis resistance in Litopenaeus vannamei by supplementation of biomastered silver nanoparticles by *Bacillus subtilis*. J. Nanomed. Nanotechnol. 2016:07. doi: 10.4172/2157-7439.1000352

[ref41] Suzuki M. Suzuki S. Matsui M. Hiraki Y. Kawano F. Shibayama K. (2013). Genome sequence of a strain of the human pathogenic bacterium *Pseudomonas alcaligenes* that caused bloodstream infection. Genome Announc. 1, e00919–e00913. doi: 10.1128/genomeA.00919-13 24179116 PMC3814577

[ref42] Tashkandy N. Sabban S. Fakieh M. Meier-Kolthoff J. P. Huang S. Tindall B. J.. (2016). High-quality draft genome sequence of *Flavobacterium suncheonense* GH29-5 T (DSM 17707 T) isolated from greenhouse soil in South Korea, and emended description of *Flavobacterium suncheonense* GH29-5 T. Stand. Genomic Sci. 11, 1–10. doi: 10.1186/s40793-016-0159-5 27313837 PMC4910214

[ref43] Udayangani R. M. C. Dananjaya S. H. S. Nikapitiya C. Heo G. J. Lee J. De Zoysa M. (2017). Metagenomics analysis of gut microbiota and immune modulation in zebrafish (*Danio rerio*) fed chitosan silver nanocomposites. Fish Shellfish Immunol. 66, 173–184. doi: 10.1016/j.fsi.2017.05.018 28479399

[ref44] Wang J. Wei H. He C. Wu G. Jian H. (2015). Optimization for extracted conditions of abalone viscera. J. Food Qual. 6, 3177–3185.

[ref45] Weon H.-Y. Song M.-H. Son J.-A. Kim B.-Y. Kwon S.-W. Go S.-J.. (2007). *Flavobacterium terrae* sp. nov. and *Flavobacterium cucumis* sp. nov., isolated from greenhouse soil. Int. J. Syst. Evol. Microbiol. 57, 1594–1598. doi: 10.1099/ijs.0.64935-0 17625200

[ref46] Xu J. Zeng X. Jiang N. Zhou Y. Zeng L. (2015). *Pseudomonas alcaligenes* infection and mortality in cultured Chinese sturgeon, *Acipenser sinensis*. Aquaculture 446, 37–41. doi: 10.1016/j.aquaculture.2015.04.014

[ref47] Yilmaz S. (2019). Effects of dietary blackberry syrup supplement on growth performance, antioxidant, and immunological responses, and resistance of Nile tilapia, *Oreochromis niloticus* to *Plesiomonas shigelloides*. Fish Shellfish Immunol. 84, 1125–1133. doi: 10.1016/j.fsi.2018.11.012 30414489

[ref48] Zhang Y. Yang Z. Ni J. Ma Y. Xiong H. Jian W. (2022). Toxicity and modulation of silver nanoparticles synthesized using abalone viscera hydrolysates on bacterial community in aquatic environment. Front. Microbiol. 13:3347. doi: 10.3389/fmicb.2022.968650 PMC9468672 36110292

